# Fracture or Not: An Easily Mistaken Benign Finding in a Tuberous Sclerosis Patient

**DOI:** 10.7759/cureus.57142

**Published:** 2024-03-28

**Authors:** Sana Padival, Tyler P Montgomery, Alan E Oestreich, James Banks

**Affiliations:** 1 Radiology, Nova Southeastern University Dr. Kiran C. Patel College of Osteopathic Medicine, Davie, USA; 2 Radiology, Aventura Hospital and Medical Center, Aventura, USA; 3 Radiology and Medical Imaging, Cincinnati Children’s Hospital Medical Center, Cincinnati, USA

**Keywords:** tuberous sclerosis, potential pitfall for misdiagnosis, skeletal radiographs, distal phalanx fracture, ungual fibroma

## Abstract

Tuberous sclerosis (TSC) is a rare autosomal dominant disorder that can affect multiple organ systems, including the brain, heart, lungs, and skin. Cutaneous manifestations are common, including ungual fibromas, however, these may be mistaken for other pathologies. Here, we present the case of a 14-year-old with TSC complaining of traumatic left little finger pain. Radiographic evaluation revealed cortical scalloping of the nailbed, concerning for a non-displaced fracture. Given the history of TSC, however, this defect may have also represented a periungual fibroma. The patient subsequently underwent conservative management and an eight-month radiographic follow-up showed no osseous remodeling, supporting the diagnosis of periungual fibroma. It is imperative for clinicians to understand the cutaneous manifestations of TSC to aid in proper diagnosis and avoidance of unnecessary treatment. In this case, interval follow-up confirmed the diagnosis and excluded fracture.

## Introduction

With an incidence of 1 in 6,000 live births in the United States [1], tuberous sclerosis (TSC) is a rare autosomal dominant disorder that can affect multiple systems by causing benign tumor growths in vital organs such as the brain, heart, lungs, and skin. Central nervous system (CNS) lesions are a hallmark of the disease and are found in all cases of TSC regardless of initial presentation. Mutations in tumor suppressor genes give rise to multiple hamartomas, particularly in the brain and skin. Cutaneous manifestations represent the most common clinical finding in TSC patients; however, these may be mistaken for other pathologies.

Here, a case of a 14-year-old patient with a history of TSC who was evaluated for a phalangeal fracture is discussed. An osseous manifestation of TSC, a periungual fibroma, confounded initial treatment and led to unnecessary fracture care and follow-up.

## Case presentation

**Figure 1:**
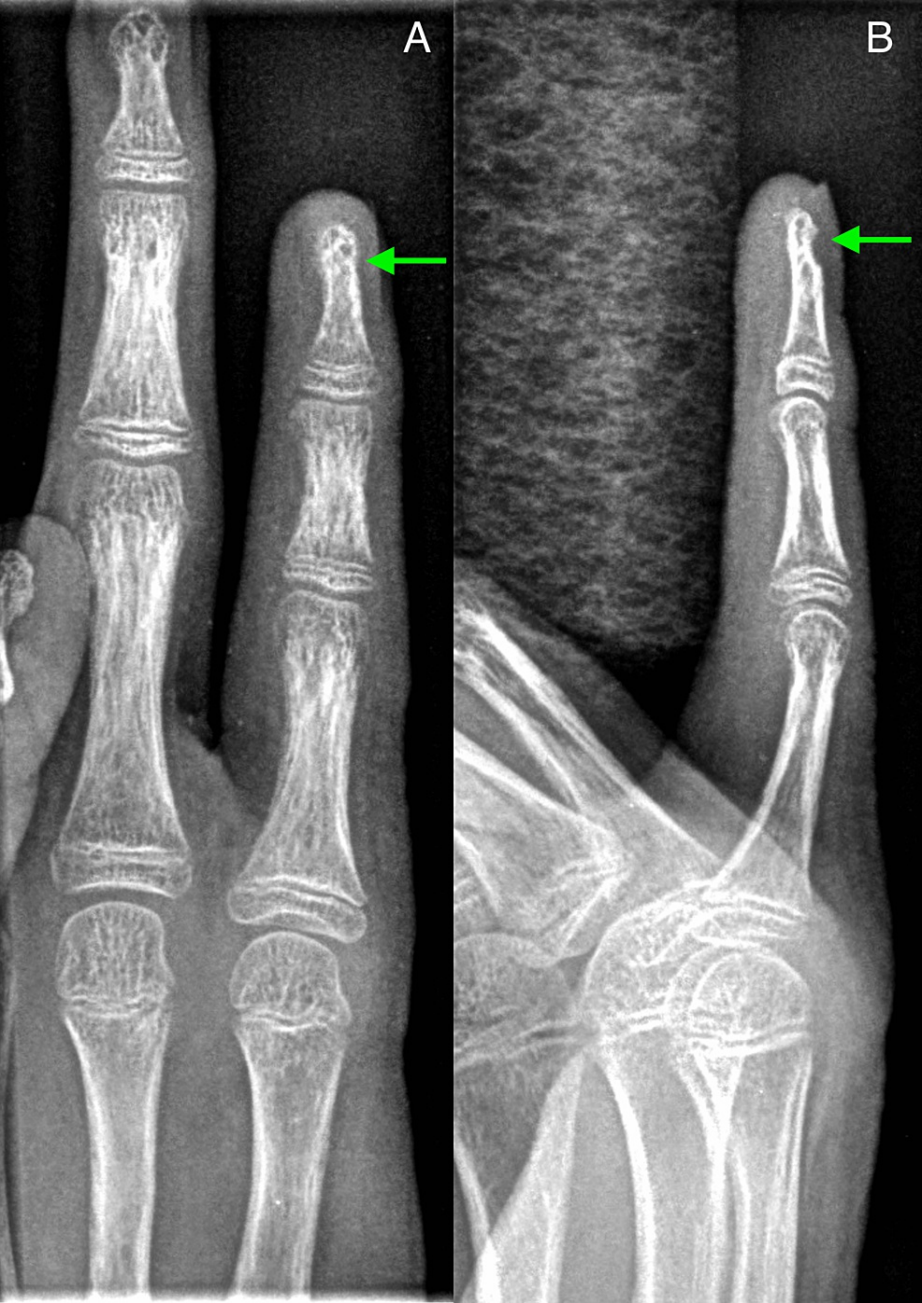
Initial radiographic evaluation of the little finger. Initial anteroposterior (A) and lateral (B) radiographs of the little finger demonstrate cortical scalloping underlying the nailbed (green arrow). Note how this is best appreciated on the lateral view and not visualized on the anteroposterior view. There is no appreciable soft tissue edema or radio-opaque foreign body.

A 14-year-old patient with a history of TSC presented with a traumatic left little finger injury, with concern for fracture. Initial radiographic evaluation revealed cortical scalloping in the region of the nailbed of the little finger (Figure 1). The remaining visualized osseous structures were intact, without evidence of physeal injury, soft tissue edema, or an unexpected radio-opaque foreign body. Based on the patient’s history and presentation, a TSC-associated periungual fibroma was considered; however, an acute traumatic fracture could not be completely excluded. Although favored to represent a benign finding, subsequent follow-up was recommended to ensure stability. The patient underwent conservative management and an eight-month radiographic follow-up showed no changes in the scalloped defect and no evidence of osseous remodeling, supporting the diagnosis of a periungual fibroma, rather than acute traumatic fracture.

## Discussion

TSC is a relatively rare autosomal dominant disorder characterized by multiple dermatological and CNS manifestations, including cortical tubers and giant cell astrocytomas, which can induce seizure activity [2]. The condition results from a loss of function of two genes, *TSC1 *and *TSC2*, which make up an inhibitory protein in the mammalian target of rapamycin signaling cascade responsible for cell proliferation [1]. This protein is part of the TSC complex, whose disruption leads to system-wide benign tumor formation of cardiac rhabdomyomas, angiofibromas, and periungual fibromas. Cutaneous lesions are one of the most common and early visible signs, and hence, have been incorporated into the 2012 TSC major diagnostic criteria for screening patients [3]. Commonly screened lesions include Shagreen patches, fibromas, and hypomelanotic macules. The TSC major diagnostic criteria include periungual fibromas, also known as Koenen tumors, requiring the presence of two or more of these lesions for definitive diagnosis [3], as fibromas can develop after nailbed trauma.

Fibromas commonly present on the hands or feet and may be asymptomatic or painful. Fibromas secondary to trauma commonly present on the fifth toe. They appear as smooth, red, or skin-colored papules along the nailbed in approximately 20% of TSC patients [4]. In a patient lacking a diagnosis of TSC, common differentials for these lesions include nailbed trauma, infection, foreign body reaction, plantar warts, histiocytosis X, and enchondroma. If findings and history are equivocal, histologic sampling will be diagnostic, showing prominent vascular and collagen bundles [4,5]. Interposed between the vascular space are densely packed bundles of vertically oriented collagen which extend through both superficial and deep skin layers, sometimes through the hypodermis to the level of the underlying cortical bone which can result in a scalloped contour.

In this case, a patient with a history of TSC was evaluated for a phalangeal fracture and had a periungual fibroma nearly mistaken as a non-displaced fracture. Such misdiagnosis can lead to short-term disability for patients, including non-weight-bearing activity, casting, splinting, or possible evaluation for surgical intervention such as percutaneous pinning. It is imperative for clinicians to understand the cutaneous manifestations of TSC patients to aid in proper diagnosis and avoidance of invasive or unnecessary treatment. In this patient’s case, interval follow-up confirmed the diagnosis, excluding fracture.

## Conclusions

TSC commonly presents with nailbed deformities known as periungual fibromas, which can cause scalloping of the underlying cortical bone. When evaluating such patients for trauma or fracture, these benign cortical defects may be mistaken for acute traumatic fracture or bone defects and lead to unnecessary testing, intervention, or clinician consultation.
